# Macro‐ and microelements in pumpkin seed oils: Effect of processing, crop season, and country of origin

**DOI:** 10.1002/fsn3.995

**Published:** 2019-04-06

**Authors:** Nenad Martinec, Sandra Balbino, Jasminka Dobša, Vesna Šimunić‐Mežnarić, Saša Legen

**Affiliations:** ^1^ Bioinstitut d.o.o. Čakovec Croatia; ^2^ Faculty of Food Technology and Biotechnology University of Zagreb Zagreb Croatia; ^3^ Faculty of Organization and Informatics University of Zagreb Varaždin Croatia

**Keywords:** macroelements, microelements, origin, processing, pumpkin seed oil

## Abstract

Macro‐ and microelements in the samples of virgin and cold pressed pumpkin seed oils produced in Croatia through two consecutive crop seasons were determined by inductively coupled plasma–optical emission spectroscopy (ICP‐OES). Croatian oils were also compared to oils from Slovenia and Austria in order to assess differences in the element content. Magnesium, potassium, calcium, sodium, selenium, and iron were the dominant elements in all pumpkin seed oils. Their amounts together with barium, strontium, manganese, copper were up to ninefold higher (*p* ≤ 0.05) in virgin compared to cold pressed pumpkin seed oils. These differences occur due to the different processing conditions which include salt addition, heat treatment, and higher degree of equipment ware out during virgin pumpkin seed oil production. As the sodium level increases with the addition of salt, virgin pumpkin seed oil could be considered its hidden source and producers should pay attention to the amount added. Contents of cobalt, copper, selenium, and thallium significantly differed (*p* ≤ 0.05) between the two crop seasons. Principal component analysis revealed clear differences between samples with different origin that can be explained by the specifics in the production processes of each country. In comparison with Austrian and Slovenian, Croatian pumpkin seed oils had significantly lower contents of sodium, potassium, calcium, magnesium, and tin while bismuth and selenium were higher.

## 1 INTRODUCTION

Pumpkin (*Cucurbita pepo *L.) seeds, used for the oil production, are a rich source of various nutrients such as fatty acids, vitamins (A and E), carotenoids, phytosterols, and squalene (Applequist, Avula, Schaneberg, Wang, & Khan, 2006; Fruhwirth & Hermetter, 2007). The intense green color of pumpkin seed oil indicates large amounts of protochlorophyll (Kreft, Zorec, Janes, & Kreft, 2009; Kreft & Kreft, 2007). Due to its positive biological effects, the consumption of pumpkin seed oil has been increasing in a number of countries, especially Austria, Slovenia, Hungary, and Croatia. As a recognition of their quality, pumpkin seed oils from Slovenia and Austria have been granted the status of protected designation of origin as Slovenian Štajerska and Prekmurje pumpkin seed oil and Styrian pumpkin seed oil, respectively (Council Regulation (EC) No 510/2006, 2006).

Along with the traditional virgin pumpkin seed oil, pressed from the roasted pumpkin seed dough which is prepared with the addition of salt and water, cold pressed pumpkin seed oil has recently appeared on the market (Rabrenovic, Dimic, Novakovic, Tecevic, & Basic, 2014).

The quantity of nutritional ingredients in pumpkin seeds depends primarily on the pumpkin type as well as the climatic and agrotechnical conditions. Furthermore, their amount in pumpkin seed oil is influenced by processing conditions applied during the production process (Cindric, Zeiner, & Steffan, 2007; Seymen, Uslu, Turkmen, Al Juhaimi, & Ozcan, 2016). In comparison with cold pressed oil, virgin pumpkin seed oil contains larger concentrations of phenolic compounds and has better oxidative stability, while cold pressed oil has higher content of tocopherols and sterols (Nakić et al., 2006).

Pumpkin seeds are also a source of potassium, zinc, manganese, selenium, and copper (Glew et al., 2006) as well as magnesium, calcium, sodium, and phosphorus, which content changes based on the cultivar and growth conditions (Fruhwirth & Hermetter, 2007; Schuster, Zipse, & Marquard, 1983). Cindric et al. (2007) investigated the mineral composition of edible (olive, pumpkin, sunflower, sesame, hazelnut, soya, rice, and grapeseed) oils and found that pumpkin seed oil together with hazelnut oil had the highest levels of magnesium and calcium, while the highest content of nickel, iron, potassium, sodium, and zinc was found in the pumpkin seed oil.

On the other hand, Joebstl, Bandoniene, Meisel, and Chatzistathis (2010) compared the impact of geographical origin of pumpkin seeds on mineral composition of rare earth elements in pumpkin seed oil and concluded that, according to their concentrations, it is possible to distinguish pumpkin seed oils from Styria, Lower Austria, and China using discriminant analysis. However, possible influence of the production processes specifics related to the country of origin was not investigated.

Since it is known that the concentration of mineral substances in oils is mostly affected by production conditions and climatic conditions of a particular cultivation year (Schuster et al., 1983), this paper was aimed to determine and compare the quantities of macro‐ and microelements in the samples of virgin and cold pressed pumpkin oils produced in 2011 and 2012 in northern Croatia. Also to get insight into the processing parameters specific to the country of origin, Croatian oils were compared with the ones for oils from Slovenia and Austria.

## 2 MATERIALS AND METHODS

### 2.1 Pumpkin seed oils

Pumpkin seed oil samples from Croatia, Slovenia, and Austria were used in this study. Croatian oils had been collected from various producers during the exhibition of pumpkin seed oils of north‐western Croatia in 2011 and 2012, as well as from the producers that had submitted their pumpkin seed oils to the laboratory of Bioinstitut d.o.o. Čakovec for food safety analysis. The oils from Slovenia were purchased in Slovenian supermarkets in 2012. The Austrian oils were purchased directly from local producers in the surroundings of Bad Radkersburg in 2012. The research in this paper was thus conducted on a total of 73 pumpkin seed oil samples. In 2011, 15 virgin oils and 4 cold pressed oils from Croatia were analyzed. In 2012, analyses were conducted on 31 virgin oils and 8 cold pressed oils from Croatia as well as 9 virgin oils from Slovenia and 6 from Austria.

### 2.2 Reagents

All the standards used in the analyses fulfilled the Atomic Spectroscopy Standard purity level. For ICP analysis, a multielement standard solution VIII: Al, B, Ba, Be, Bi Ca, Cd, Co, Cr, Cu, Fe, Ga, K, Li, Mg, Mn, Na, Ni, Pb, Se, Sr, Te, Tl, Zn obtained by Merck (Darmstadt, Germany) was used. Trace metals III: Ba, Ca, Mo, Na, K, Mg were purchased by Perkin Elmer Life and Analytical Sciences (Shelton, USA). Tin, antimony, silver, vanadium, silicon, and lanthanum standards for atomic absorption were obtained from Carlo Erba Reagenti SpA (Rodano, Italy). The acids used for the digestion of samples in the microwave oven and for diluting the standard solutions fulfilled the proanalysis purity level. Nitric acid 65% and hydrochloric acid ≥37% were obtained from Carlo Erba Reagenti SpA (Rodano, Italy) and Sigma‐Aldrich Chemie GmbH (Steinheim, Germany), respectively.

### 2.3 Microwave digestion

Mars Xpress (CEM Corporation, Matthews, USA) microwave oven was used for microwave digestion of samples. Digestion was made according to the application note for fats and oils. Visual inspection of samples after digestion showed no signs of precipitation, turbidity, or residual carbon, and no precipitate was present on the filter after sample filtration. In the process of digestion, 1 g of the sample is put into a Teflon tube, to which 5 ml of 65% nitric acid and 1 ml of 36.5% hydrochloric acid are added. The microwave digestion was performed in four stages (first stage: *p* = 75 W, *t* = 100°C, *t*_retention_ = 10 min; second stage: *p* = 150 W, *t* = 150°C, *t*_retention_ = 10 min; third stage: *p* = 225 W, *t* = 190°C, *t*_retention_ = 20 min; fourth stage: *p* = 300 W, *t* = 230°C, *t*_retention_ = 30 min). The rate of temperature increase in all stages was 15 min. After cooling, the samples were quantitatively transferred into volumetric vessels of 50 ml and diluted with deionized water of <0.05 μS/cm conductivity.

### 2.4 ICP‐OES method

The metals in sample solutions were quantified by inductively coupled plasma–optical emission spectrometry (ICP‐OES Optima 7000; Perkin Elmer, USA), in accordance with the official method (HRN EN ISO 11885, 2010). The method is based on the measurement of radiation emission by an optical spectroscopic technique. The sample is dispersed into aerosol and introduced in the plasma, where atom excitation and radiation emission occur. The radiation emission produces characteristic line emission spectra. On the basis of signal intensity, the measurement element concentration is calculated. The settings of the ICP‐OES Optima 8000 instrument are as follows: RF power 1,300 W; plasma argon flow rate 8 ml/min; auxiliary flow rate 0.2 L/min; nebulizer argon flow rate 0.8 ml/min; pump rate 1.5 ml/min; standard torch; cross flow nebulizer; axial plasma viewing; two replicates for each analysis run; sample delay time of 30 s.

For qualitative identification of particular elements, wavelengths (nm) that are characteristic for each element were applied and are shown in Table 1. Solutions containing highest concentrations of the calibration range for each element were used as calibration check solutions for the daily routine instrument monitoring.

**Table 1:** Results of the ICP‐OES method validation

Element/analytical lines^a^	Wavelength (nm)	Limits of detection (mg/kg)	Limits of quantification (mg/kg)	Recovery (%)	Calibration range (mg/kg)
Li I	670.784	0.300	0.900	99.2	5–50
Na I	589.592	0.087 (ax); 0.365 (rad)	0.261 (ax); 1.095 (rad)	92.8	5–100
K I	766.490	0.211 (ax); 0.397 (rad)	0.633 (ax); 1.191 (rad)	93.4	5–100
Ba II	233.527	0.009	0.028	98.8	5–100
Ca II	317.933	0.089 (ax); 0.21 (rad)	0.267 (ax); 0.630 (rad)	97.0	5–100
Mg I	285.213	0.032 (ax); 0.044 (rad)	0.096 (ax); 0.132 (rad)	98.7	5–100
Be II	313.107	0.005	0.015	91.3	0.5–250
Sr II	407.771	0.005	0.015	95.82	2.5–50
Cr II	267.716	0.013	0.039	100.2	0.5–250
Mo II	202.031	0.058	0.174	102.2	2.5–250
Zn I	213.857	0.035	0.105	98.9	2.5–250
Co II	228.616	0.010	0.030	96.5	0.5–250
Ni II	221.648	0.021	0.063	103.7	0.5–250
Mn II	257.610	0.003	0.009	99.2	0.5–250
Cu I	324.752	0.027	0.081	98.7	0.5–250
Fe II	238.204	0.010	0.030	99.0	0.5–250
Ag I	328.068	0.018	0.054	101.5	2.5–250
V II	309.310	0.067	0.201	93.6	2.5–250
Cd I	228.802	0.005	0.015	97.5	0.5–50
Pb II	220.353	0.065	0.195	103.2	2.5–250
Se I	196.026	0.189	0.567	108.5	5–100
Sn II	189.927	0.257	0.771	105.7	2.5–250
Al I	396.153	0.069	0.207	101.9	0.5–250
B I	249.677	0.223	0.669	101.9	5–100
Bi I	223.061	0.005	0.015	99.5	5–50
Tl II	190.801	0.845	2.535	114.29	5–500
Ga I	417.206	0.005	0.015	101.95	5–50
La II	398.852	0.025	0.075	103.5	5–50
Te I	214.281	0.290	0.870	98.00	5–50
Sb I	206.836	0.381	1.143	97.2	2.5–250
Si I	251.611	0.180	0.540	102.0	5–500
As I	188.979	0.172	0.516	102.8	2.5–250

a I‐atomic lines; II‐ionic.

### 2.5 Method validation

The validation of the method included the following parameters: linearity, limit of detection (LOD), limit of quantification (LOQ), and recovery. The limits of detection and quantification were determined by a tenfold measurement of a blind‐trial solution at a particular wavelength characteristic for each metal. The standard deviation was calculated and the triple value of the obtained standard deviation (3 × *SD*) was taken as the limit of detection, while the ninefold value of the obtained standard deviation (9 × *SD*) was taken as the limit of quantification. For calcium, magnesium, sodium, and potassium, the values of the axial (ax) as well as the radial (rad) view are provided owing to detector saturation that occurs during axial determination with higher concentrations of those elements, which are common in food samples, so there is often a switch to the radial view. For all the other elements, the values are expressed only for the axial view (Table 1). The recovery of the method was determined by spiking (prior to digestion) a pumpkin seed oil sample of known base values with the standard solutions in three different concentrations representing the lowest, middle, and highest concentrations of the calibration range. Recovery is expressed as the middle ratio of the calculated (measured value reduced by base value) and the actual added amount of elements (Table 1).

### 2.6 Statistical analysis

The concentrations of micro‐ and macroelements in the examined pumpkin seed oils were determined and expressed as the mean values, relative standard deviations, and the ranges of results obtained for a particular subgroup of samples. XLStat 2017 (Addinsoft, Paris, France) package was used to perform the analysis of variance (ANOVA) and post hoc Tukey test, determine the correlations between individual elements, and conduct the principal component analysis (PCA).

## 3 RESULTS AND DISCUSSION

Subgroups of Croatian pumpkin seed oil samples were formed based on the production process (virgin and cold pressed) (Table 2) and production year (2011 and 2012) (Table 3). Oils from 2012 were also compared based on the country of origin (Croatia, Austria, and Slovenia) (Table 4). Among determined elements, the following groups were defined as follows: (a) alkali and alkaline earth metals; (b) transition metals; and (c) post‐transition metals, metalloids, and lanthanum. Out of 32 measured elements, 14 elements (Ag, As, Be, Cd, La, Li, Mo, Ni, Pb, Sb, Si, Te, V, and Zn) were below quantification limits shown in Table 1 in all analyzed samples.

**Table 2:** Concentrations of elements (mg/kg) in Croatian virgin and cold pressed pumpkin seed oils subgroups

Element	Virgin (*n* = 46)		Cold pressed (*n* = 12)	
	Mean ± *SD*	Range	Mean ± *SD*	Range
Li	<LOQ	<LOQ	<LOQ	<LOQ
Na*	7.969 ± 5.836	<LOQ–20.219	0.894 ± 2.402	<LOQ–8.264
K*	22.517 ± 14.413	5.298–83.713	8.358 ± 14.376	1.312–49.446
Ba*	0.063 ± 0.025	<LOQ–0.142	0.040 ± 0.015	<LOQ–0.061
Ca*	13.421 ± 8.678	2.334–39.684	3.181 ± 2.830	0.597–11.356
Mg*	35.469 ± 17.552	11.856–83.941	6.461 ± 8.317	<LOQ–31.501
Be	<LOQ	<LOQ	<LOQ	<LOQ
Sr*	0.010 ± 0.020	<LOQ–0.050	<LOQ	<LOQ
Cr	0.013 ± 0.091	<LOQ–0.619	<LOQ	<LOQ
Mo	<LOQ	<LOQ	<LOQ	<LOQ
Zn	<LOQ	<LOQ	<LOQ	<LOQ
Co	0.010 ± 0.016	<LOQ–0.042	0.003 ± 0.010	<LOQ–0.034
Ni	<LOQ	<LOQ	<LOQ	<LOQ
Mn*	0.190 ± 0.099	0.060–0.408	0.029 ± 0.060	<LOQ–0.216
Cu*	0.744 ± 0.859	<LOQ–3.821	0.070 ± 0.104	<LOQ–0.219
Fe*	2.698 ± 4.475	<LOQ–25.408	0.345 ± 0.633	<LOQ–2.167
Ag	<LOQ	<LOQ	<LOQ	<LOQ
V	<LOQ	<LOQ	<LOQ	<LOQ
Cd	<LOQ	<LOQ	<LOQ	<LOQ
Pb	<LOQ	<LOQ	<LOQ	<LOQ
Se*	2.783 ± 0.639	1.803–5.209	2.312 ± 0.765	1.491–4.103
Sn*	0.768 ± 0.497	<LOQ–1.703	1.232 ± 0.477	<LOQ–1.716
Al	0.835 ± 0.972	<LOQ–6.696	1.177 ± 1.268	0.236–4.410
B	0.194 ± 0.505	<LOQ–1.540	0.122 ± 0.421	<LOQ–1.460
Bi*	0.027 ± 0.033	<LOQ–0.100	0.056 ± 0.027	<LOQ–0.100
Tl*	0.288 ± 0.422	<LOQ–1.054	<LOQ	<LOQ
Ga	0.005 ± 0.029	<LOQ–0.195	0.032 ± 0.074	<LOQ–0.189
La	<LOQ	<LOQ	<LOQ	<LOQ
Te	<LOQ	<LOQ	<LOQ	<LOQ
Sb	<LOQ	<LOQ	<LOQ	<LOQ
Si	<LOQ	<LOQ	<LOQ	<LOQ
As	<LOQ	<LOQ	<LOQ	<LOQ

Notes

<LOQ: below the limit of quantification.

* Significant influence of production process (*p* ≤ 0.05).

**Table 3:** Concentrations of elements (mg/kg) in Croatian pumpkin seed oils from 2011 and 2012 subgroups

Element	2011 (*n* = 19)		2012 (*n* = 39)	
	Mean ± *SD*	Range	Mean ± *SD*	Range
Li	<LOQ	<LOQ	<LOQ	<LOQ
Na	8.084 ± 6.806	<LOQ–20.219	5.737 ± 5.544	<LOQ–19.169
K	23.747 ± 16.679	1.312–58.318	17.562 ± 14.539	1.407–83.713
Ba	0.060 ± 0.016	0.036–0.095	0.057 ± 0.028	<LOQ–0.142
Ca	12.622 ± 11.997	0.597–39.684	10.659 ± 6.949	1.324–27.231
Mg	34.310 ± 24.801	3.100–83.941	27.108 ± 16.936	<LOQ–62.154
Be	<LOQ	<LOQ	<LOQ	<LOQ
Sr	0.021 ± 0.025	<LOQ–0.050	0.002 ± 0.009	<LOQ–0.049
Cr	0.033 ± 0.142	<LOQ–0.619	<LOQ	<LOQ
Mo	<LOQ	<LOQ	<LOQ	<LOQ
Co*	<LOQ	<LOQ	0.013 ± 0.017	<LOQ–0.042
Ni	<LOQ	<LOQ	<LOQ	<LOQ
Mn	0.152 ± 0.121	<LOQ–0.408	0.159 ± 0.110	<LOQ–0.370
Cu*	0.216 ± 0.008	0.192–0.222	0.794 ± 0.937	<LOQ–3.821
Fe	1.479 ± 1.734	<LOQ–5.995	2.568 ± 4.837	<LOQ–25.408
Ag	<LOQ	<LOQ	<LOQ	<LOQ
V	<LOQ	<LOQ	<LOQ	<LOQ
Cd	<LOQ	<LOQ	<LOQ	<LOQ
Pb	<LOQ	<LOQ	<LOQ	<LOQ
Zn	<LOQ	<LOQ	<LOQ	<LOQ
Se*	2.990 ± 0.563	2.131–4.103	2.538 ± 0.700	1.491–5.209
Sn	1.007 ± 0.306	<LOQ–1.545	0.795 ± 0.594	<LOQ–1.716
Al	0.628 ± 0.254	0.294–1.122	1.041 ± 1.235	<LOQ–6.696
B	<LOQ	<LOQ	0.266 ± 0.576	<LOQ–1.540
Bi	0.033 ± 0.032	<LOQ–0.097	0.033 ± 0.035	<LOQ–0.100
Tl*	0.697 ± 0.380	<LOQ–1.054	<LOQ	<LOQ
Ga	<LOQ	<LOQ	0.016 ± 0.052	<LOQ–0.195
La	<LOQ	<LOQ	<LOQ	<LOQ
Te	<LOQ	<LOQ	<LOQ	<LOQ
Sb	<LOQ	<LOQ	<LOQ	<LOQ
Si	<LOQ	<LOQ	<LOQ	<LOQ
As	<LOQ	<LOQ	<LOQ	<LOQ

Notes

<LOQ: below the limit of quantification.

* Significant influence of crop season (*p* ≤ 0.05).

**Table 4:** Concentrations of elements in Croatian, Slovenian and Austrian virgin pumpkin seed oils from 2012

Element	Croatia (*n* = 31)		Slovenia (*n* = 9)		Austria (*n* = 6)	
	Mean ± *SD*	Range	Mean ± *SD*	Range	Mean ± *SD*	Range
Li	<LOQ	<LOQ	<LOQ	<LOQ	<LOQ	<LOQ
Na*	6.881 ± 5.509^a^	<LOQ–19.169	45.846 ± 58.041^b^	<LOQ–130.234	149.420 ± 12.066^c^	136.062–164.641
K*	20.608 ± 15.051^a^	5.298–83.713	42.397 ± 26.643^b^	<LOQ–83.627	85.470 ± 22.249^c^	62.414–112.618
Ba*	0.062 ± 0.029^a^	<LOQ–0.142	0.068 ± 0.027 ^a,b^	0.043–0.131	0.086 ± 0.031^b^	0.058–0.132
Ca*	12.372 ± 6.795^a^	2.334–27.231	9.475 ± 6.057^a^	<LOQ–18.688	33.628 ± 14.895^b^	19.915–52.245
Mg*	32.173 ± 14.533^a^	11.856–62.154	32.146 ± 19.542^b^	<LOQ–61.571	115.440 ± 22.528^b^	91.079–142.785
Be	<LOQ	<LOQ	<LOQ	<LOQ	<LOQ	<LOQ
Sr*	0.002 ± 0.010^a^	<LOQ–0.049	0.059 ± 0.043^b^	<LOQ–0.148	0.045 ± 0.030^b^	0.018–0.082
Cr	<LOQ^a^	<LOQ	0.005 ± 0.014^a^	<LOQ–0.041	<LOQ^a^	<LOQ
Mo	<LOQ	<LOQ	<LOQ	<LOQ	<LOQ	<LOQ
Zn	<LOQ	<LOQ	<LOQ	<LOQ	<LOQ	<LOQ
Co	0.015 ± 0.017^a^	<LOQ–0.042	0.011 ± 0.017^a^	<LOQ–0.035	0.012 ± 0.018^a^	<LOQ–0.038
Ni	<LOQ	<LOQ	<LOQ	<LOQ	<LOQ	<LOQ
Mn	0.190 ± 0.098^a^	0.060–0.370	0.182 ± 0.085^a^	0.070–0.337	0.564 ± 0.209^b^	0.368–0.839
Cu*	0.998 ± 0.901^a^	<LOQ–3.821	0.009 ± 0.028^b^	<LOQ–0.083	<LOQ^b^	<LOQ
Fe	3.123 ± 3.455^a^	<LOQ–25.408	2.664 ± 1.431^a^	0.213–4.564	2.648 ± 1.236^a^	1.550–4.333
Ag	<LOQ	<LOQ	<LOQ	<LOQ	<LOQ	<LOQ
V	<LOQ	<LOQ	<LOQ	<LOQ	<LOQ	<LOQ
Cd	<LOQ	<LOQ	<LOQ	<LOQ	<LOQ	<LOQ
Pb	<LOQ	<LOQ	<LOQ	<LOQ	<LOQ	<LOQ
Se*	2.697 ± 0.683^a^	1.803–5.209	1.968 ± 0.337^b^	1.681–2.653	2.135 ± 0.537^a,b^	1.591–3.150
Sn*	0.635 ± 0.550^a^	<LOQ–1.703	1.367 ± 0.243^b^	1.132–1.962	1.930 ± 0.458^b^	1.119–2.304
Al	0.943 ± 1.213^a^	<LOQ–6.696	0.796 ± 1.314^a^	<LOQ–4.128	1.203 ± 2.351^a^	<LOQ–5.993
B	0.287 ± 0.620^a^	<LOQ–1.540	<LOQ^a^	<LOQ	<LOQ^a^	<LOQ
Bi*	0.027 ± 0.036^a^	<LOQ–0.100	<LOQ^b^	<LOQ	<LOQ^b^	<LOQ
Tl	<LOQ	<LOQ	<LOQ	<LOQ	<LOQ	<LOQ
Ga	0.008 ± 0.038^a^	<LOQ–0.195	<LOQ^a^	<LOQ	<LOQ^a^	<LOQ
La	<LOQ	<LOQ	<LOQ	<LOQ	<LOQ	<LOQ
Te	<LOQ	<LOQ	<LOQ	<LOQ	<LOQ	<LOQ
Sb	<LOQ	<LOQ	<LOQ	<LOQ	<LOQ	<LOQ
Si	<LOQ	<LOQ	<LOQ	<LOQ	<LOQ	<LOQ
As	<LOQ	<LOQ	<LOQ	<LOQ	<LOQ	<LOQ

Notes

<LOQ: below the limit of quantification.

Different letters in the same row indicate significant differences between samples (Tukey test, *p* ≤ 0.05).

* Significant influence of country of origin (*p* ≤ 0.05).

### 3.1 Alkali and alkaline earth metals

Elements from this group, especially magnesium, potassium, calcium, and sodium, play important roles in many physiological functions of the human body. Since their intake levels have been connected to many different conditions and diseases such as hypertension, heart attack, and various gastrointestinal cancers, it is considered that homeostasis of these elements greatly contributes to the overall well‐being (Blaine, Chonchol, & Levi, 2015; Glasdam, Glasdam, & Peters, 2016). According to estimates of World Health Organization (2014), chronic noncommunicable diseases, for example, arterial hypertension, diabetes, obesity, cardiac, renal and pulmonary diseases, and cancer, are responsible for 86% of premature deaths in Europe. Excessive intake of kitchen salt is considered to be one of the most important risk factors for the occurrence of all these diseases. Sodium is, however, also found hidden in foods such as whole wheat bread and processed cereals which are generally considered “healthy” (Magriplis et al., 2011).

According to the mean values obtained though this study (Table 2), virgin pumpkin seed oil is particularly rich in magnesium (35.469 mg/kg), potassium (22.517 mg/kg), calcium (13.421 mg/kg), and sodium (7.969 mg/kg); however, wide ranges related to their contents in the individual samples were noted. Low quantities of barium and strontium (0.063 and 0.010 mg/kg, respectively) were also found. These results are somewhat different than those found in the study on commercial pumpkin seed oil samples by Juranovic, Breinhoelder, and Steffan (2003). They reported considerably lower values of potassium (below the limit of detection of their method, i.e., 0.248 mg/kg) as well as a considerably higher level of sodium (35.1 mg/kg). The concentrations of magnesium (44.7 mg/kg) were higher and calcium (5.80 mg/kg) lower than the means obtained in our research; however, they were in the here determined range.

On the other hand, Cindric et al. (2007) reported values of calcium, potassium, magnesium, and sodium (16.9, 45.3, 16.4 and 20.6 mg/kg, respectively) in Croatian pumpkin seed oils which were more similar to the ones obtained in this research.

Statistical analysis applied to the obtained results had shown that production process causes significant differences in the content of dominant alkali and alkaline earth metals. In cold pressed oils, the mean concentration of sodium was about 9 times lower (*p* ≤ 0.001), magnesium and calcium were about 5 times lower (*p* ≤ 0.001), and the concentration of potassium was 2.5 times lower (*p* = 0.003) than in the virgin oils. Somewhat lower amounts of barium (0.040 mg/kg, *p* = 0.004) were present in cold pressed oils, while strontium was not detected. Higher concentrations of these elements in comparison with cold pressed oils subgroup can be explained by the addition of salt in the virgin pumpkin seed oil production process. Namely, according to the specifications of Slovenian Štajerska and Prekmurje pumpkin seed oil (SI/PGI/0105/01361) and Styrian pumpkin seed oil (AT/PGI/0017/1460), salt is added to pumpkin seed dough usually in the amount of 1%–2%. As kitchen salt, besides sodium chloride, also contains certain amounts of calcium, magnesium, and potassium, the amounts of these elements are also higher (De la Guardia & Garrigues, 2015). In addition, heat applied during roasting could have attributed to the increase of the dominant elements due to the thermal degradation of the cell structures as it was already reported for roasted peanuts and sesame seeds and oils (Aljuhaimi & Özcan, 2018; Hassan, 2013).

In their studies, Juranovic et al. (2003) compared industrially processed to laboratory‐extracted pumpkin seed oils and reported that salt addition during pumpkin seed dough mixing increases the concentrations of sodium, calcium, potassium, magnesium, and phosphorus in the industrial samples. Similar to our results, their contents in laboratory‐extracted oils were ~10 times lower than in the industrially processed oils made from roasted seeds. Mitić et al. (2018) also analyzed the content of potassium in Serbian pumpkin seed oils and found similar values for roasted oils; however, values for cold pressed oils were much higher than the ones from this research.

The results of the analysis for this group of elements in pumpkin seed oils from different crop seasons are similar and do not show statistically significant differences. As there are no previous investigations done on pumpkin seed oils, these results can be compared to findings of Bell, Rakow, and Downey (1999) who explored the differences in the mineral composition of three types of seeds of Brassica species grown in the western part of Canada between 1990 and 1993. The analyses of concentrations of calcium and magnesium in their work showed no statistically significant differences between the seeds pertaining to different production year.

### 3.2 Transition elements

In Croatian virgin pumpkin seed oils investigated in this study (Table 2), the highest average concentrations of transition elements were obtained for iron (2.698 mg/kg), followed by copper (0.744 mg/kg) and manganese (0.190 mg/kg). Lower concentrations were established for chromium (0.013 mg/kg) and cobalt (0.010 mg/kg) while molybdenum, nickel, silver, vanadium, zinc, and cadmium were below quantification in all samples. The mean values of manganese, copper, and iron were lower (*p* ≤ 0.001) in the cold pressed oils, while chrome was not detected. As it was mentioned, the differences in the concentrations of these elements can be partly explained by the residues from the mentioned salt addition (De la Guardia & Garrigues, 2015). However, heavy metals originating from the machines ware out are more likely the cause especially considering that iron, chromium, and manganese are the main constituents of stainless steel which is the main food processing equipment construction material (Kamerud, Hobbie, & Anderson, 2013). Namely, roasting of pumpkin seed dough during virgin oil production process takes place in the steel frying pans which are equipped with metal scrapers that prevent the dough sticking to and burning on the pan bottom. Due to the needed close contact of the pan and the scraper, abrasion, which can lead to higher iron, chromium, and manganese contents in the virgin seed oil, occurs.

In their study, Juranovic et al. (2003) obtained considerably higher values of elements from this group. The concentration of chrome (6.8 mg/kg) was hundred times higher than the mean for virgin pumpkin seed oils in this research, while the concentrations of copper and iron were about 10 times higher (12.1 mg/kg for copper; 16.1 mg/kg for iron; and 3.2 mg/kg for zinc). The average concentrations of cadmium and molybdenum were 1.7 mg/kg and 0.80 mg/kg, respectively. Nickel, manganese, and cobalt were not detected. Furthermore, Mitić et al. (2018) reported even higher values of iron (up to 67.4 mg/kg). Roasted pumpkin seed oils analyzed in their work seemingly contained higher amounts of iron, even though the statistical significance was not established. In the research of Cindric et al. (2007), manganese, cobalt, and chrome were not detected in pumpkin seed oil samples, the concentration of nickel was 6.1 mg/kg, while the levels of copper (0.40 mg/kg) were comparable to those obtained in this research.

In the oils from 2012, the average concentration of iron was 2.568 mg/kg and apparently higher than in 2011 (1.479 mg/kg). However, although there were individual samples in 2012 in which the concentrations of iron were much higher (25.41 and 16.17 mg/kg) than in 2011, this difference was not significant. On the other hand, the average value of copper in 2012 was more than four times higher than the one obtained in the previous year (0.794 and 0.216 mg/kg respectively, *p* ≤ 0.01), while cobalt was detected only in 2012 (*p* ≤ 0.001). Chrome was not detected in 2012 samples; however, there were no differences at the 0.05 level of significance just like in the case of manganese. Bell et al. (1999) and Gąstoł and Domagała‐Świątkiewicz (2009) state that pH is a remarkably important factor which has an impact on the absorption of minerals from the soil to the plant. The soil pH can be determined, among others, by the type and amount of mineral fertilizers as well as the weather conditions, that is, the type and amount of precipitation. According to the Statistical Yearbook of the Republic of Croatia (Ostroški, 2011), a total of 1,200.3 mm of precipitation and the average annual temperature of 10.4°C was recorded for the Varaždin measurement station in 2010 (crop season of pumpkin seed oils produced in 2011). In that year, 31 occurrences of acid rain were detected by the Zagreb measurement station. In 2011, the situation was fairly different. In that year in the area of the Varaždin measurement station, 481.2 mm of precipitation was recorded, with the average annual temperature of 11.2°C and only eight occurrences of acid rain detected by the Zagreb measurement station (Ostroški, 2012). Possible impact of the aforementioned climatic conditions, that is, a smaller amount of precipitation in 2011 is evident in higher concentrations of copper, cobalt, and vanadium in comparison with their concentrations in 2012.

### 3.3 Post‐transition metals, metalloids, and lanthanum

Gfrerer and Zischka (2003) analyzed pumpkin seed oil samples from the Austrian province of Styria using the ICP mass spectrometer after microwave digestion and detected varying concentrations of cadmium, arsenic, lead, and mercury from this element group. As these elements are considered toxic heavy metal contaminants, their levels in food have to be closely monitored (Zhu, Fan, Wang, Qu, & Yao, 2011).

The elements from this group found in the highest average amount in the virgin oils (Table 2) were selenium (2.783 mg/kg), aluminum (0.835 mg/kg), tin (0.768 mg/kg), and thallium (0.288 mg/kg). While bismuth (0.027 mg/kg) was considerably low, cadmium, lead, zinc, boron, gallium, lanthanum, tellurium, antimony, silicon, and arsenic were not detected. Compared to cold pressed oils, concentration of selenium (*p* ≤ 0.05) was higher in virgin pumpkin seed oils, while tin (*p* ≤ 0.001) and bismuth (*p* ≤ 0.01) were lower. Also, thallium was present in virgin oils but it was not detected in any of the cold pressed samples.

Juranovic et al. (2003) found that concentrations of both lead and aluminum were below the detection limits of their method (0.166 and 0.920 mg/kg, respectively). Similar findings regarding the concentration of lead being below the detection limit of 0.22 mg/kg were found by Cindric et al. (2007), who also reported the concentration of aluminum was 1.50 mg/kg which is somewhat higher than the levels detected in our study. Maximum amount of lead allowed in oils is set to 0.1 mg/kg by the Commission Regulation (EC) No 1881/2006 (2006). As lead was not detected in any of the samples, the results of this study show satisfactory safety related to this parameter.

Kreft, Stibilj, and Trkov (2002) analyzed the concentration of selenium in pumpkin seed oils and seeds and concluded that pumpkin seed oil could not be considered a source of selenium. The levels of selenium for oils in their research were below the method limit (0.001 mg/kg). On the other hand, Mitić et al. (2018) determined concentration of selenium in pumpkin seed oils up to 1.21 mg/kg, which indicates that the amount of that element can vary greatly. Because oils from this study and previous ones have been performed on the pumpkin seed samples originating from different areas, this variation in the content of selenium can be explained by differences of its concentrations in the soil. Roca‐Perez et al. (2010) analyzed content of selenium in Spanish soils and found it ranges from 0.06 to 1.51 mg/kg, and it is dependent on the pollution, soil acidity, and clay content.

Considering the differences in the content of elements from this group caused by crop season, the concentrations of selenium were significantly lower in 2012 than in the previous year and thallium was not detected in any of the 2012 samples.

### 3.4 Comparison between macro‐ and microelements in virgin pumpkin seed oils according to country of origin

Results for the contents of analyzed elements in virgin pumpkin seed oils from Croatia, Slovenia, and Austria are shown in Table 4. Unlike Croatian oils, in which it was only the fourth highest, in Slovenian and Austrian oils, sodium was the dominant mineral with 149.420 and 45.846 mg/kg, respectively. Average potassium values have shown similar behavior with 85.470, 42.397, and 20.206 mg/kg measured in Austrian, Slovenian, and Croatian oils, respectively. Results of the performed Tukey post hoc test have also shown significant differences in sodium and potassium content between all the countries of origin. Contents of magnesium were also significantly higher in Slovenian and Austrian oils, but they did not differ between each other, while calcium was the highest in Austrian oils. Consequently, as it was mentioned that the origin of potassium in virgin pumpkin seed oil is the salt added during its production, it can be assumed that Austrian and some Slovenian producers add higher amounts of salt during the preparation of pumpkin seed dough for roasting. Also, as the intensity of heat treatment causes better transfer of elements from seeds to oil, Austrian as well as Slovenian producers of virgin pumpkin seed oil could be using higher temperatures and/or longer roasting times in comparison with Croatian producers. Significant differences were also noted in the contents of barium and strontium from the alkali and alkaline earth metals group. In transition element group, only copper was significantly higher in Croatian oils, while in the post‐transition metals, metalloids, and lanthanum group, significant differences were noted for the selenium, tin, and bismuth which were again higher in Slovenian and Austrian oils. Since the differences between countries considering the four elements present in the highest amounts in the samples are most notable for sodium and considering that kitchen salt is added in the process of oil production, it can be assumed that there are differences in the amount of salt used in the production process in each of the three countries.

Distribution of various trace elements in seed oils is dependent on their content in the soil. Some of these elements, such as rare earth elements, derive from the underlying rock and are therefore related to the geographical origin (Bandoniene, Zettl, Meisel, & Maneiko, 2013). On the other hand, environmental conditions, that is, air and soil pollution can significantly influence the content of elements, especially heavy metals in the edible oils (Angelova, Ivanova, & Ivanov, 2005).

Grabmann (2009) conducted a multielement analysis of pumpkin seed oils from Austria, Slovenia, and Croatia as well as Serbia, Hungary, and China. Among alkali and alkaline earth metals, author included all the elements that are also investigated in this study, except for lithium and beryllium. Concentrations of particular elements that were determined in their study were ten or more fold different according to the country of origin. The reported results closely correspond with those in this research. In Grabmann's study (2009), the concentrations of manganese (0.08 mg/kg) and iron (0.82 mg/kg) in Croatian oils were somewhat lower than in our research, while the values for Slovenian and Austrian oils were fairly similar. Among the other analyzed elements, it is worthwhile mentioning copper and zinc, whose values for Austrian and Slovenian oils were fairly higher than the ones obtained in this research.

To further investigate the noted differences between the countries of origin, principal components analysis (PCA), which enables efficient reduction of data dimensionality, was used. PCA is often applied to examine the interrelationships between macro‐ and microelements of various foodstuff (Frías, Conde, Rodríguez‐Bencomo, García‐Montelongo, & Pérez‐Trujillo, 2003; Kment et al., 2005) as it identifies common dimensions within which parameters can be classified and their correlations with the country of origin can be established. For the purpose of this study, the PCA was run on the concentrations of macro‐ and microelements in virgin pumpkin seed oils. Macro‐ and microelements that had been detected in pumpkin seed oils were used as variables, while the samples of virgin pumpkin seed oils from Croatia, Slovenia, and Austria represented the cases. A total of 63 cases and 10 variables, chosen based on the existence of significant differences between countries, were included in the analysis. The aim of the PCA was to establish whether the samples can be distinguished by the country of origin on the basis of the obtained values of macro‐ and microelements. The first factor (F1) accounted for 47.15% of the total data variance. Among the other factors, only the second factor (F2) for which eigenvalue was greater than 1 was further considered. These first two factors accounted for 62.96% of the total data variance.

**Figure 1:**
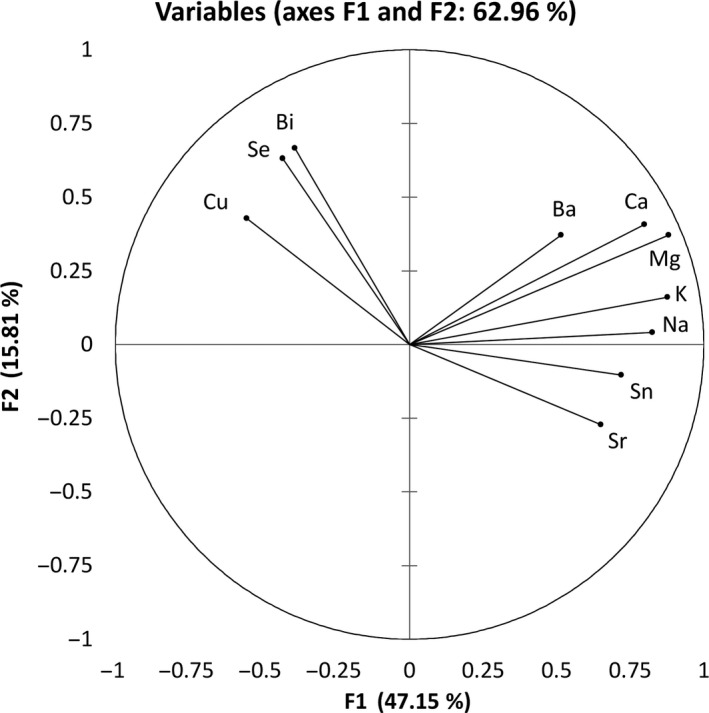
Projection of the active variables determined in virgin pumpkin seed oils from different country of origin on the factorial plane

According to the projection of variables (Figure 1), F1 is strongly positively correlated with magnesium (*r* = 0.881), potassium (*r* = 0.877), sodium (*r* = −0.826), calcium (*r* = 0.798), and tin (*r* = 0.720). Its moderate positive correlation was established with strontium (*r* = 0.651) and barium (*r* = 0.515). F1 is also in moderate negative correlation with copper (*r* = −0.553). On the other hand, F2 has a moderate positive correlation selenium (*r* = 0.666) and bismuth (*r* = 0.631).

**Figure 2:**
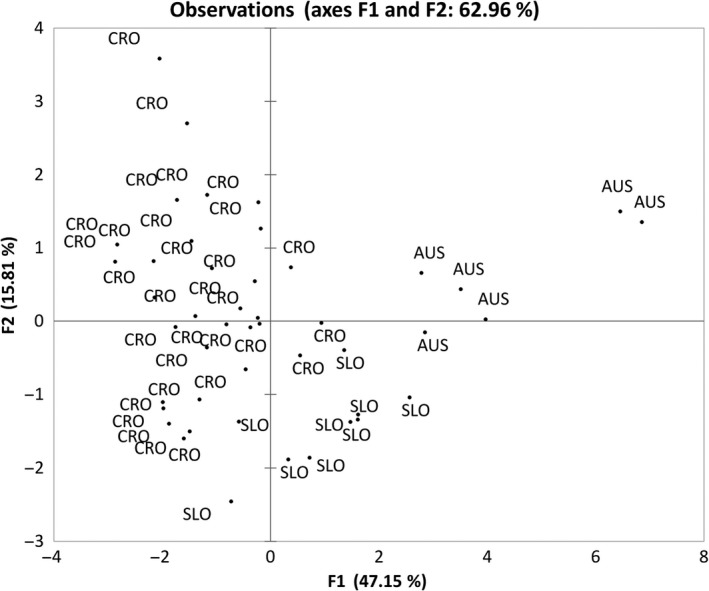
Projection of virgin oil samples with different country of origin on the factorial plane (AUS: Austrian oils; CRO: Croatian oils; SLO: Slovenian oils)

In addition, Figure 2 shows the projection of samples of virgin pumpkin seeds oils in the plane of factors 1 and 2. Considering the aim of this research, it is clearly evident that all Austrian and most of the Slovenian oils are grouped in the first and second quadrant, that is, in the area in which the first factor was assigned positive values. In addition, if we compare Slovenian and Austrian oils, it is notable that all the Austrian oils are located further to the right in relation to Slovenian oils, which is a consequence of a higher concentration of elements that are strongly positively correlated with F1—magnesium, potassium, sodium, calcium, and tin. Only three Croatian oils are on the right side of coordinate system while the rest have negative values for F1 and are dispersed across second and third quadrant showing. Such a distribution can be explained by differences in the contents of elements for which positive correlations F2 were established, that is, bismuth and selenium. Consequently, owing to the aforementioned reasons, in the two‐factor plane grouping of samples with the same country of origin is evident and confirms specifics in the applied production processes.

## 4 CONCLUSION

According to the results of this investigation, it can be concluded that the content of macro‐ and microelements in pumpkin seed oils varies significantly under the influence of processing conditions, crop seasons, and countries of origin. Magnesium, potassium, calcium, and sodium were dominant elements in both oil types; however, their content was significantly higher in virgin pumpkin seed oils compared to cold pressed. These differences occur due to the addition of kitchen salt for pumpkin seed dough preparation and also because of the heat‐induced damage to cells and release of cellular material during roasting. Even though the quantities of sodium found in pumpkin seed oil are relatively low and do not pose a direct health threat, with the growing evidence on the importance of detection of hidden salt sources, reported findings of this research surely call for attention. On the other hand, crop season characteristics, that is, the amount of precipitation, were accounted for the minor but significant differences in the content of copper and cobalt. In addition, comparison of samples in regard to the country of origin revealed that sodium, potassium, calcium, and magnesium contents were significantly higher in Slovenian and Austrian oils than in Croatian oils. These differences confirm the existence of applied processing specifics, mainly related to the amounts of salt addition.

## ETHICAL STATEMENT

The study did not involve any human or animal experimentation.

## CONFLICT OF INTEREST

The authors have declared no conflict of interest.
